# White matter integrity of the medial forebrain bundle and attention and working memory deficits following traumatic brain injury

**DOI:** 10.1002/brb3.608

**Published:** 2016-12-20

**Authors:** Jacqueline A. Owens, Gershon Spitz, Jennie L. Ponsford, Alicia R. Dymowski, Nicholas Ferris, Catherine Willmott

**Affiliations:** ^1^ School of Psychological SciencesMonash UniversityMelbourneVic.Australia; ^2^ Monash‐Epworth Rehabilitation Research CentreEpworth HealthCareMelbourneVic.Australia; ^3^ Monash Institute of Cognitive and Clinical NeurosciencesMonash UniversityMelbourneVic.Australia; ^4^ Monash Biomedical ImagingMonash UniversityMelbourneVic.Australia

**Keywords:** attention, medial forebrain bundle, traumatic brain injury

## Abstract

**Background and Objective:**

The medial forebrain bundle (MFB) contains ascending catecholamine fibers that project to the prefrontal cortex (PFC). Damage to these fibers following traumatic brain injury (TBI) may alter extracellular catecholamine levels in the PFC and impede attention and working memory ability. This study investigated white matter microstructure of the medial MFB, specifically the supero‐lateral branch (slMFB), following TBI, and its association with performance on attention and working memory tasks.

**Method:**

Neuropsychological measures of attention and working memory were administered to 20 moderate‐severe participants with TBI (posttraumatic amnesia *M* = 40.05 ± 37.10 days, median time since injury 10.48 months, range 3.72–87.49) and 20 healthy controls. Probabilistic tractography was used to obtain fractional anisotropy (FA) and mean diffusivity (MD) values for 17 participants with TBI and 20 healthy controls.

**Results:**

When compared to controls, participants with TBI were found to have significantly lower FA (*p* < .001) and higher MD (*p* < .001) slMFB values, and they were slower to complete tasks including Trail Making Task‐A, Hayling, selective attention task, *n*‐back, and Symbol Digit Modalities Test.

**Conclusion:**

This study was the first to demonstrate microstructural white matter damage within the slMFB following TBI. However, no evidence was found for an association of alterations to this tract and performance on attentional tasks.

## 1 Introduction

Attention and working memory deficits are prevalent cognitive impairments following moderate to severe traumatic brain injury (TBI; Ponsford & Willmott, 2004; Willmott, Ponsford, Hocking, & Schonberger, 2009). These deficits adversely affect individuals’ ability to work, socialize and function in everyday life (Bercaw, Hanks, Millis, & Gola, 2010; Draper, Ponsford, & Schönberger, 2007). The dopamine (DA) system is thought to play a key role in persistent cognitive impairment following TBI, including attention deficits (for review see Bales, Wagner, Kline, & Dixon, 2009). Elucidating TBI‐induced disruptions to the DA system and whether they are associated with attention deficits may assist in identifying those most likely to benefit from pharmacological interventions.

Experimental models (Brozoski, Brown, Rosvold, & Goldman, 1979; Montaron, Bouyer, Rougeul, & Buser, 1982) and clinical studies specifically implicate the DA system in attention and working memory function (Clark, Geffen, & Geffen, 1986, 1987a,b, 1989). Dysfunction of DA circuitry has previously been found in other clinical groups demonstrating attentional deficits, particularly Attention Deficit/Hyperactivity Disorder (see del Campo, Chamberlain, Sahakian, & Robbins, 2011 for review), with administration of DA agonists found to ameliorate attentional impairments (Arnsten, 2011; Nieoullon, 2002; Solanto, 1998; Willmott & Ponsford, 2009). TBI causes widespread damage that may disrupt the DA system, potentially leading to attention deficits.

Alterations to the DA system have previously been identified in TBI populations. Reduced DA levels have been found in cortical areas post‐TBI (McIntosh, Yu, & Gennarelli, 1994). Alterations to the DA transporter protein have been identified in brain regions associated with attentional function following TBI, including the frontal cortices (Yan, Kline, Ma, Li, & Dixon, 2002), and the striatum (Donnemiller et al., 2000). This is believed to be secondary to disruptions to catecholamine pathways via diffuse axonal injury (DAI; Donnemiller et al., 2000). DAI is a common pathology in TBI that leads to a cascade of events, including denervation and degeneration of nerve terminals (Büki & Povlishock, 2006; Johnson, Stewart, & Smith, 2013). Disruptions to DA signaling seen after TBI, and the associated attention impairments, may be somewhat attributable to alteration to DAergic pathways caused by DAI. Investigation into alterations to DA pathways following TBI and association with attention deficits, however, is lacking.

The medial forebrain bundle (MFB) is an important pathway within the DA system. It contains ascending catecholamine fibers that innervate the frontal cortices (Coenen, Panksepp, Hurwitz, Urbach, & Mädler, 2012; Coenen, Schlaepfer, Maedler, & Panksepp, 2011; Coenen et al., 2009). Due to its major role in the brain reward systems, past research has generally focused on the MFB in relation to affective and addiction disorders (Alcaro & Panksepp, 2011; Bracht, Doidge, Keedwell, & Jones, 2015; Coenen et al., 2009, 2012; Wise, 2005). In humans, the supero‐lateral branch (slMFB) of the MFB connects the ventral tegmental area (VTA) to the anterior limb of the internal capsule, the ventral striatum, and nucleus accumbens, before terminating in the prefrontal cortex (PFC; Coenen et al., 2009, 2011, 2012). Given that alterations to the slMFB may have secondary consequences for extracellular DA levels, and potentially underpin attentional impairments, investigation of the changes to the slMFB following TBI is important.

Diffusion tensor imaging (DTI) is a magnetic resonance technique that provides an indication of the microstructural damage to white matter pathways by measuring water diffusivity, resulting in measures of fractional anisotropy (FA) and mean diffusivity (MD) (Le & Gean, 2009; Provenzale, 2010). DTI has been successfully used to identify alterations to white matter microstructure associated with poorer attentional performance post‐TBI. Reduced speed of information processing has been associated with microstructural alterations in frontal white matter, cingulum bundle, inferior longitudinal fasciculus, thalamic projections, and corpus callosum (Arenth, Russell, Scanlon, Kessler, & Ricker, 2013; Little et al., 2010; Spitz, Maller, O'Sullivan, & Ponsford, 2013; Wilde et al., 2011). Attention span has been found to be associated with white matter microstructure within thalamic projections post‐TBI (Little et al., 2010). Additionally, the superior longitudinal fasciculus, corona radiata, and corpus callosum are implicated in attentional control (Arenth et al., 2013; Spitz et al., 2013).

The absence of the MFB on DTI neuroanatomical atlases makes it less accessible for investigation than other more prominent white matter tracts. It was not until 2009 that the MFB was first depicted using DTI (Coenen et al., 2009). In their initial DTI deterministic fiber tracking investigation of the MFB, Coenen et al. (2009) tracked the MFB by placing a region of interest (ROI) seed in the ventral midbrain. In a subsequent investigation, Coenen et al. (2012) again used deterministic tractography and tracked the MFB by placing a single ROI seed in the ipsilateral VTA. Bracht et al. (2015), employed a similar ROI to depict both the infero‐medial (imMFB) and slMFB using probabilistic tractography. Additionally, Anthofer et al. (2015) compared three different ROI pairs for deterministic fiber tracking of the MFB. The most reliable and convincing results were found when using the ipsilateral VTA and nucleus raphe dorsalis ROI pair. Using this method, Anthofer et al. (2015) found similar results to Bracht et al. (2015), Coenen et al. (2009, 2012), replicating the author's anatomical description of the MFB, as well as the seed regions used.

The aim of this study was to: (1) explore the white matter microstructure of the slMFB in a TBI population in comparison with controls, and (2) explore the association between slMFB white matter microstructure and attentional function following TBI.

## 2 Method

### 2.1 Participants

Twenty participants with a history of moderate to very severe TBI (15 male) and 20 healthy controls (12 male) were recruited from the Acquired Brain Injury Rehabilitation service at Epworth HealthCare, Melbourne, Australia. Healthy controls were recruited from the general public and explicitly matched to TBI participants during recruitment. The groups did not differ significantly with respect to age, years of education, estimated IQ, or gender (Table 1). For the TBI group, median time since injury was 10.48 months (interquartile range [IQR] = 10.83 months, range 0–87.5 months). Forty percent of participants underwent MRI and cognitive testing on the same day (for the remainder median time lag between assessment and scan = 25 days, IQR = 62.25 days, with the assessment done first in 86% of cases). Individuals were excluded if they had an inadequate understanding of English, insufficient cognitive ability or physical disabilities preventing completion of the tasks, previous history of treatment for psychiatric illness, past neurological disorder, history of treatment for drug or alcohol dependence, diagnosis of attention deficit disorder prior to injury, or magnetic resonance (MR) contraindications. Cause of injury included motor‐vehicle accident (60%), bicycle or pedestrian accidents involving motor vehicles (25%), falls (10%), and one participant was involved in an equestrian accident (5%). Mean duration of post traumatic amnesia (PTA) was 40.05 days (*SD* = 37.10 days, range 0–142 days). With regard to GCS, 15% were classified as mild (GSC 13–15), 5% were moderate (GCS 9–12), 75% were severe (GCS 3–8), and 5% were not recorded. In terms of PTA duration, 50% of TBI participants had a very severe injury (PTA > 4 weeks), 35% a severe injury (PTA 1–4 week), 10% a moderate injury (PTA 1–7 days), and one participant (5%) a complicated mild injury (PTA < 24 hours with changes on computed tomography [CT] imaging; Arlinghaus, Shoaib, & Price, 2005). All TBI participants demonstrated evidence of damage on CT brain scans (Table 1).

**Table 1:** Demographic and brain pathology of the TBI and control groups

	TBI (*n *=* *20)	Controls (*n *=* *20)	*p*‐Value	Effect size^a^
	Mean (*SD*)	Mean (*SD*)		
Gender	15	*12*	.31	0.32
Age (years)	39.05 (16.45)	33.45 (11.72)	.22	−0.40
Years of education	13.58 (2.53)	13.43 (2.67)	.86	−0.06
Estimated FSIQ	108.83 (8.81)	107.71 (5.87)	.64	−0.15
Brain pathology (%)				
Contusion	8 (40)	**–**		
Diffuse axonal injury	3 (15)	**–**		
Subarachnoid hemorrhage	8 (20)	**–**		
Subdural hemorrhage	10 (50)	**–**		
Epidural hemorrhage	2 (10)	**–**		
Intracranial hemorrhage	3 (15)	**–**		
Interventricular hemorrhage	3 (15)	**–**		
Hematoma	4 (20)	**–**		
Abscesses	1 (5)	**–**		
Petechial hemorrhages	3 (15)	**–**		

FSIQ, Full Scale Intelligence Quotient; TBI, traumatic brain injury.

a Effect size is Cohen's *D*.

Two TBI participants were excluded from the imaging data analysis due to severe pathology preventing accurate depiction of the MFB. One TBI participant had large focal frontal lobe lesions, whereas the other participant had significant hydrocephalus. One participant was excluded from the analysis due to inconsistencies in DICOM images rendering them incompatible with the neuroimaging analysis, leaving 17 for analysis. Two further TBI participants were excluded from the correlation analysis as their cognitive assessment and MRI were more than a month apart, rendering the association between DTI metrics and cognitive performance potentially invalid, resulting in *n* = 15. TBI participants (*n* = 20) were included in all comparisons for cognitive tasks, with the exception of one color‐blind participant for the computerized selective attention task (SAT) analysis.

### 2.2 Procedure

This study was approved by Monash University Human Research and Epworth HealthCare Ethics Committees. Written informed consent was obtained from all participants. Once they emerged from PTA according to their treating neuropsychologist, measured by daily administration of the Westmead PTA Scale (Shores, Marosszeky, Sandanam, & Batchelor, 1986), participants were invited to undergo a neuropsychological assessment of attention and working memory as well as a brain MRI scan. Prior to enrolment, all participants underwent CT scans as a part of routine assessment and treatment at the acute hospital. Results from CT scans were reported by radiologists at the respective hospitals.

### 2.3 Neuropsychological measures

#### 2.3.1 The National Adult Reading Test

The National Adult Reading Test (NART; Nelson, 1982) is a reading test that consists of 50 irregularly spelt words and was used to estimate pre‐morbid IQ.

#### 2.3.2 The Symbol Digit Modalities Test

The Symbol Digit Modalities Test (SDMT; Smith, 1991) has previously been used to show reduced psychomotor processing speed in a TBI sample (Ponsford & Kinsella, 1992). Participants have 90 s to decode a series of symbols.

#### 2.3.3 The computerized selective attention task

The SAT (Ziino & Ponsford, 2006) has two conditions, the simple selective attention task (SSAT) and complex selective attention task (CSAT). The CSAT assessed a higher working memory load as participants are required to retain additional verbal rules. TBI patients have been found to respond more slowly and make significantly more errors than controls on this task (Willmott & Ponsford, 2009).

#### 2.3.4 The Ruff 2&7 selective attention task

The Ruff 2&7 SAT (Ruff & Allen, 1995) is a pen and paper cancelation task with two conditions, automatic speed (ASRS), controlled speed (CSRS). Participants canceled the digits 2 and 7 among either letters or numbers, with the former being an automatic retrieval condition and the latter requiring controlled search and working memory abilities.

#### 2.3.5 The *n*‐back

The *n*‐back (Perlstein et al., 2004) has been found to be sensitive to working memory deficits in a TBI sample (Perlstein et al., 2004). Participants were required to correctly match the letter presented on the screen with the letter presented 0, 1, and 2 screens back.

#### 2.3.6 The Hayling Sentence Completion Test from the Hayling and Brixton Tests

The Hayling Sentence Completion Test (Burgess & Shallice, 1996) has two sets of 15 sentences with the last word missing. Hayling A (response initiation) required participants to provide a word that completes the sentence as quickly as possible. In Hayling B (response inhibition), participants were required to complete the sentences with a word that is completely unrelated to the sentence. The tasks measure speed of initiation and response inhibition and has been found to be sensitive to change post‐TBI (Draper & Ponsford, 2008).

#### 2.3.7 Trail Making Test—Parts A and B

The Trail Making Test (TMT; Reitan & Wolfson, 1985), required participants to connect 25 numbers in ascending order (Trails A), and to switch between 13 numbers and 12 letters in sequence (Trails B), as quickly as possible while maintaining accuracy. It measures processing speed, divided attention, and mental flexibility and has been shown to differentiate individuals with TBI from healthy control participants (Spitz, Ponsford, Rudzki, & Maller, 2012).

#### 2.3.8 Digit Span subtest of the Wechsler Adult Intelligence Scale—fourth editions

Digits backwards, forwards, and sequencing (Wechsler, 1997), was used to assess participants’ immediate auditory attention span and working memory capacity. Digit span backwards has previously been shown to be sensitive to changes in attention after TBI (Chan, 2000; Kinsella et al., 1996).

### 2.4 Neuroimaging acquisition

Neuroimaging was performed on a Siemens Magnetom Skyra 3 Tesla MRI scanner using a 32 channel head coil (Siemens Medical Imaging, Erlangen, Germany). A 3D T1‐weighted MPRAGE sequence was acquired in the sagittal orientation (TI 900 ms, TR 1540 ms, TE 2.57 ms, resolution 256 × 256 × 176, flip angle 9°, FOV 250 mm, slice thickness 1.00 mm [176 slices]). A DTI sequence was acquired (TR = 10,900, TE = 101,64 diffusion encoding directions, number of excitations = 1, slice thickness = 2.0 mm (64 slices), field of view = 256 mm, matrix = 128 × 128, in‐plane = 2.0 × 2,0 mm, *b* value = 2,000 s/mm^2^).

#### 2.4.1 Medial forebrain bundle tractography

**Figure 1:**
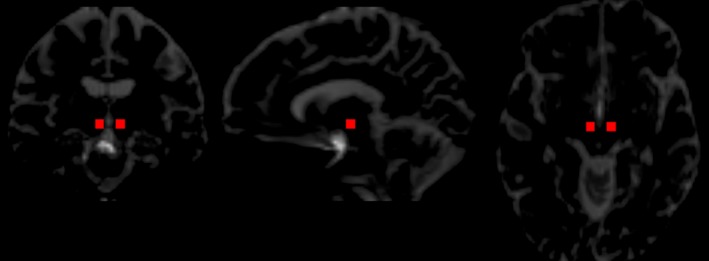
Red square indicates the initial seed location used for probabilistic tractography in a single participant

Tractography of the MFB was conducted in MRtrix version 3 (RRID:SCR_006971; J‐D Tournier, Brain Research Institute, Melbourne, Australia, http://www.mrtrix.org/; Tournier, Calamante, & Connelly, 2012). Diffusion‐weighted images initially underwent eddy‐current correction in FSL version 5.0.8 (RRID:SCR_002823). The standard DWI processing was then undertaken in MRtrix, including estimating the response function before conducting the Constrained Spherical Deconvolution based on the previously obtained response function. Using probabilistic tractography, the slMFB was tracked for each individual on the DWI image in subject‐native space. The MFB seed regions outlined by Coenen et al. (2012) were used in this study. The ipsilateral VTA was used as the seed point, with the anterior margin being the mammillary body/mammillo‐thalamic tract, the lateral margin the medial margin of the substantia nigra, and the posterior margin the red nucleus (see Figure 1). The first 10 resulting slMFB tracts were visually inspected by an experienced neuroradiologist NF, and authors GS and JO inspected the remainder to ensure correct depiction of the slMFB. Each individual's slMFB tract was converted to a slMFB mask, weighted on streamline length. Mean FA and MD images were generated from the diffusion‐weighted images in subject native space using an iteratively reweighted linear least‐squares solver (Veraart, Sijbers, Sunaert, Leemans, & Jeurissen, 2013). FA and MD values for each participant were extracted by overlying the slMFB mask on each of the corresponding images.

### 2.5 Data analysis

Using a cut off of reaction times (RTs) greater or less than two standard deviations from the mean for each group, 3% of *n*‐back RTs across three conditions as well as 8% SAT RTs across two conditions were removed from the analysis, similar to previous RT studies (Willmott et al., 2009). Univariate outliers were defined as *Z*‐score > 3.29 (*p* < .001, two‐tailed test). Two outliers in the Hayling Test were identified (one TBI and one healthy control for number of errors) and assigned a score 1 unit greater than the next most extreme score (Tabachnick & Fidell, 2007). Independent sample *t* tests were undertaken to compare groups for normally distributed test variables. Nonparametric test Mann–Whitney *U* was used to analyze error data for the SDMT, 2&7 accuracy data, TMT, Hayling Test, and errors, missed responses for the *n*‐back and SAT. Pearson's correlations were run to explore the relationship between white matter microstructure of the MFB and performance on attention and working memory tasks, controlling for age, years of education and estimated Intelligence Quotient (FSIQ). To control for Type I error rate, Bonferroni adjustments were made for multiple comparisons.

## 3 Results

### 3.1 Neuropsychological performance

Using a Bonferroni adjusted α level of .004, when compared to control participants, TBI participants were found to complete fewer items on the SDMT, cancel fewer targets in both the controlled search and automatic detection condition of the Ruff 2&7, and were significantly slower to complete the TMT‐A as well as the timed aspects of the Hayling Test (Table 2). With regard to computerized tasks, they were slower to respond during all conditions of the *n‐*back and SAT, than controls (Table 2). No significant differences were found between the TBI and control group on any condition of Digit Span.

**Table 2:** Means, standard deviations, significance, and effect sizes for performance on attention tasks for TBI and control groups

Task	Measure	TBI (*n = *20)	Control (*n *=* *20)	*p*‐Value	Effect size^a^
		Mean (*SD*)	Mean (*SD*)		
Digit Span	DSF RS	11.35 (2.56)	11.40 (2.87)	.94	0.02
	DSB RS	9.25 (2.95)	8.85 (2.76)	.66	0.14
SDMT	Number correct	44.30 (9.58)	58.10 (10.20)	<.001	1.40
TMT	TMT‐A time (s)	39.60 (15.02)	23.05 (7.50)	<.001	1.40
	TMT‐B time (s)	83.50 (39.65)	59.90 (22.01)	.027	0.74
Hayling	Initiation Time (s)	16.70 (16.29)	4.65 (4.49)	.004	1.01
	Inhibition Time (s)	49.05 (44.30)	10.25 (9.39)	.001	1.21
2&7	ASRS	109.50 (30.54)	158.80 (24.03)	<.001	1.80
	CSRS	102.30 (22.33)	138.35 (20.23)	<.001	1.70
SAT^b^	SSAT RT (ms)	863.81 (178.04)	654.48 (98.26)	<.001	1.45
	CSAT RT (ms)	1595.12 (366.32)	1205.40 (209.97)	<.001	1.31
*n*‐back	0‐back RT (ms)	775.19 (186.97)	611.57 (129.71)	.003	1.02
	1‐back RT (ms)	866.88 (189.67)	690.30 (150.32)	.002	1.03
	2‐back RT (ms)	1111.63 (256.70)	788.86 (190.89)	<.001	1.43

DSF, digit span forward; RS, raw score; DSB, digit span backward; TMT, Trail Making Test; ASRS, Automatic Speed Raw Score; CSRS, Controlled Speed Raw Score; SAT, selective attention task; SSAT, simple selective attention task; RT, reaction time; CSAT, complex selective attention task; SDMT, Symbol Digit Modalities Test; TBI, traumatic brain injury.

a Effect size is Cohen's *D*.

b SAT TBI (*n* = 19).

Mann–Whitney *U* tests revealed that number of errors on the Hayling SDMT, TMT‐A, or TMT‐B, or accuracy on any condition of the Ruff 2&7 did not differ between groups (Table 3). In terms of the computerized tasks, the groups did not differ with regard to number of errors or misses on any condition of the SAT or the *n*‐back (Table 3).

**Table 3:** Means, standard deviations, and significance for errors score for TBI and control groups

	TBI	Control	*p*‐Value	Effect size^a^
	*M* (*SD*)	*M* (*SD*)		
SDMT				
Errors	0.65 (1.09)	0.75 (1.2)	.60	−0.09
TMT				
TMT‐A errors	0.20 (0.41)	0.25 (0.55)	.97	−0.10
TMT‐B errors	0.60 (0.75)	0.60 (0.94)	.80	0
Hayling				
Initiation errors	10.26 (16.06)	1.70 (2.39)	.08	0.75
Inhibition errors	4.21 (4.66)	1.25 (1.25)	.03	0.87
Ruff 2& 7				
AS accuracy	95.01 (4.79)	96.21 (4.02)	.48	−0.27
CS accuracy	92.10 (8.00)	92.85 (4.90)	.68	0.38
SAT^b^				
SSAT errors	0.20 (0.52)	0.05 (0.22)	.58	0.38
SSAT misses	0	0	1.00	−0.11
CSAT errors	2.40 (2.32)	2.40 (3.03)	.86	0
CSAT misses	0.30 (0.57)	0.10 (0.31)	.41	0.44
*n*‐back				
0‐back errors	1.25 (2.00)	0.25 (0.44)	.09	0.69
0‐back misses	0.65 (1.79)	0	.11	0.51
1‐back errors	2.95 (2.33)	1.40 (1.73)	.04	0.76
1‐back misses	0.85 (1.18)	0.10 (0.44)	.03	0.84
2‐back errors	6.60 (4.36)	3.25 (3.04)	.01	0.89
2‐back misses	1.70 (2.49)	0.05 (0.22)	.005	0.93

TMT, Trail Making Test; AS, automatic speed; CS, controlled speed; SAT, selective attention task; SSAT, simple selective attention task; CSAT, complex selective attention task; SDMT, Symbol Digit Modalities Test; TBI, traumatic brain injury.

a Effect size is Cohen's *D*.

b SAT TBI (*n* = 19).

### 3.2 Supero‐lateral branch of the medial forebrain bundle

**Figure 2:**
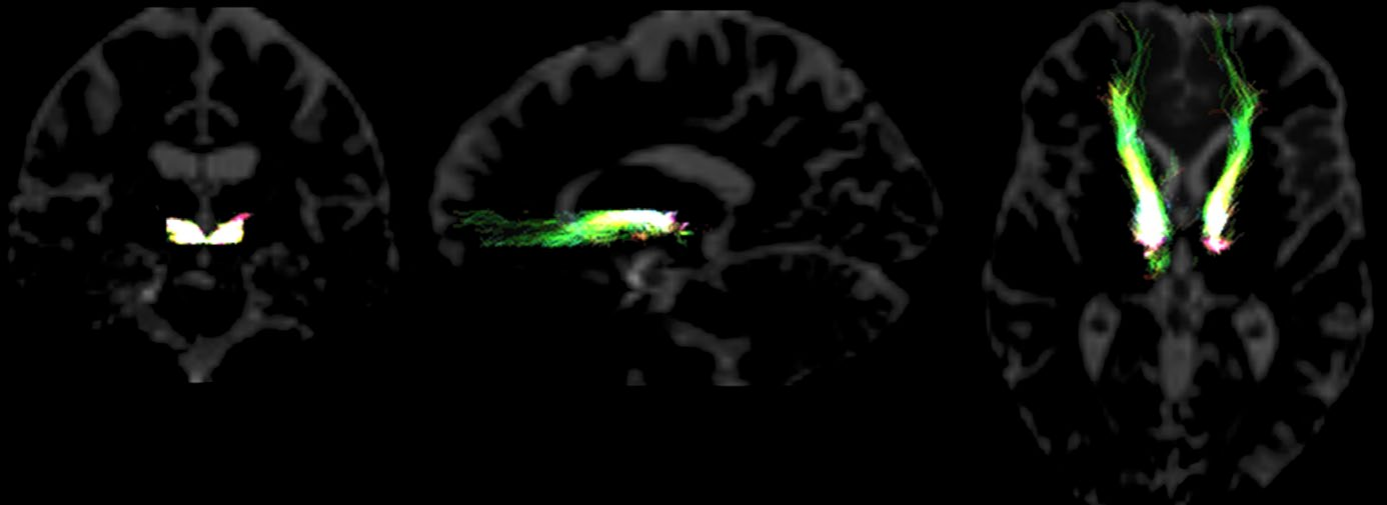
Supero‐lateral branch reconstructed for a single participant using probabilistic tractography

Visual inspection of the results approximated that found by the Anthofer et al. (2015), Bracht et al. (2015), and Coenen et al. (2009, 2012) depictions of the MFB when using the ipsilateral VTA and nucleus raphe dorsalis as ROIs. Thus, the slMFB identified follows the same path described in these previous studies—running from the seed point placed in the VTA, the fibers courses along the lateral wall of the third ventricle connecting to the nucleus accumbens and anterior limb of the internal capsule, before terminating in the inferior‐medial PFC, see Figure 2.

### 3.3 White matter microstructure alterations within the slMFB following TBI

Using a Bonferroni adjusted α level of .025, bivariate comparisons revealed that TBI participants had significantly lower FA values and significantly higher MD values within the slMFB compared to the control group (Table 4). Participants with TBI also showed a larger range of slMFB FA and MD values than controls. When controlling for age and time since injury, no significant association was found between worst GCS or PTA duration and slMFB FA or MD values. In addition, no association was found between time since injury and DTI metrics when controlling for age.

**Table 4:** Means, standard deviations, significance, and effect sizes for diffusion tensor imaging metrics for TBI and control groups

	TBI (*n = *17)		Control (*n *=* *20)		*p*‐Value	Effect size^a^
	Mean (*SD*)	Range	Mean (*SD*)	Range		
FA	0.33 (0.03)	0.256–0.371	0.37 (0.03)	0.320–0.426	<.001	−1.56
MD^b^	7.43 (0.50)	6.5–8.5	6.74 (0.29)	6.1–7.2	<.001	1.69

FA, fractional anisotropy; MD, mean diffusivity; TBI, traumatic brain injury.

a Effect size is Cohen's *D*.

b MD (×10^−4^ mm^2^/s).

Using a Bonferroni adjusted α level of *p *>* *.001, when controlling for age, years of education, and gender, no significant correlations were found between neuropsychological performance and DTI metrics for TBI or control participants (Table 5).

**Table 5:** Correlation coefficient and *p*‐values for diffusion tensor imaging metrics and cognitive measures, controlling for age, years of education, and estimated full‐scale intelligence quotient

	TBI		Control	
	FA	MD	FA	MD
SDMT				
Number correct	.171*.594*	−.495*.102*	−.172*.509*	.114*.662*
Ruff 2&7				
ASRS	−.428*.165*	.085*.792*	−.433*.083*	.294*.252*
CSRS	−.418*.176*	.129*.690*	−.300*.242*	.215*.406*
TMT				
TMT‐A (s)	−.298*.347*	.323*.306*	.025*.924*	.153*.559*
TMT‐B (s)	.162*.614*	−.220*.492*	.358*.158*	−.175*.502*
Hayling				
Initiation time (s)	−.224*.485*	−.005*.987*	−.029*.911*	−.023*.930*
Inhibition time (s)	.203*.526*	−.132*.683*	−.439*.078*	.249*.335*
Initiation errors	−.130*.686*	−.067*.836*	−.174*.504*	.156*.549*
Inhibition errors	−.002*.995*	−.125*.698*	−.458*.065*	.275*.285*
Digit span				
DSF	−.220*.492*	.595*.041*	−.039*.882*	.182*.484*
DSB	−.170*.597*	.558*.059*	.007*.978*	.069*.792*
DSS	−.243*.447*	−.023*.943*	−.270*.295*	.221*.393*
*n*‐back				
0‐back RT (ms)	−.219*.494*	.625*.030*	−.349*.170*	.071*.788*
1‐back RT (ms)	.445*.147*	−.675*.016*	−.254*.325*	−.086*.742*
2‐back RT (ms)	−.267*.401*	.446*.146*	−.274*.287*	.015*.954*
SAT^a^				
SSAT RT (ms)	.413*.182*	−.024*.940*	−.272*.290*	−.042*.873*
CSAT RT (ms)	.127*.695*	.039*.905*	.133*.611*	−.379*.134*

*p*‐values presented in italics; FA, fractional anisotropy; MD, mean diffusivity; DSF, digit span forward; RS, raw score; DSB, digit span backward; TMT, Trail Making Test; ASRS, Automatic Speed Raw Score; CSRS, Controlled Speed Raw Score; SAT, selective attention task; SSAT, simple selective attention task; RT, reaction time; CSAT, complex selective attention task; SDMT, Symbol Digit Modalities Test; TBI, traumatic brain injury.

a SAT TBI (*n* = 19).

## 4 Discussion

To the best of our knowledge, this was the first study to investigate changes to the slMFB following TBI, and their association with attention and working memory deficits. As the slMFB contains ascending catecholamine fibers, damage as a result of DAI may disrupt extracellular concentrations of catecholamines within the PFC, potentially leading to attention and working memory deficits. Previous DTI deterministic fiber tracking research has described the slMFB as splitting from the trunk of the MFB in the VTA, coursing along the lateral wall of the third ventricle, connecting to the nucleus accumbens and anterior limb of the internal capsule, before terminating in the inferior‐medial PFC (Anthofer et al., 2015; Bracht et al., 2015; Coenen et al., 2009, 2012). Using probabilistic tractography and seed regions described by Coenen et al. (2012), the slMFB was depicted as described by previous literature, replicating previous findings.

Using a well‐matched sample, TBI participants were found to have reduced FA and higher MD within the slMFB when compared to controls, indicating microstructural white matter damage caused by DAI. This is consistent with the many previous studies which have identified multiple damaged white matter pathways following TBI (see Hulkower, Poliak, Rosenbaum, Zimmerman, & Lipton, 2013 for review). Disruption to axon terminals caused by DAI may affect DA transmission (Büki & Povlishock, 2006), and downregulation of the dopamine transporter protein has been identified post‐TBI (Donnemiller et al., 2000; Yan et al., 2002). Although DA agonists have been associated with amelioration of attention deficits post‐TBI (Whyte et al., 1997, 2004; Willmott & Ponsford, 2009), the underlying disruption to the DA system is still not well understood. The current findings extend upon previous research identifying disruptions to the DA system following TBI (Bales et al., 2009; Fujinaka, Kohmura, Yuguchi, & Yoshimine, 2003; Yan et al., 2002). Research into the type and extent of damage associated with TBI is important to further our understanding of the disorder and may help to identify individuals suitable for pharmacological trials.

Contrary to our hypothesis, no significant associations were found between slMFB FA or MD values and attentional outcomes in the TBI or control group, failing to provide evidence for a role of the slMFB in attention or working memory processes. This finding is inconsistent with experimental models which have demonstrated inattentive behavior to be associated with lesioning of the mesocorticolimbic neurons (Salamone, 1991)‐ DA neurons that project through the MFB (Moore & Bloom, 1978). Additionally, the DA pathways arising from the VTA, connecting to the striatum/nucleus accumbens and terminating in the PFC are believed to be linked to attention deficits in other disorders, particularly ADHD (del Campo et al., 2011; Kharas & Dafny, 2016). Research has identified reduced FA in orbitofrontal‐striatal pathways in individuals with ADHD, however, associations with attention measures were not investigated (Schweren et al., 2016).

Given that TBI has been linked to complex, varied, and interactive pathology (Werner & Engelhard, 2007), and the attentional system itself is diffuse and complex, perhaps it is not surprising that no association was found when focusing on a single pathway. Other pathology associated with TBI may have affected areas implicated in attentional abilities, giving rise to the deficits seen in the TBI group. However, no association was identified between slMFB microstructure and attentional performance in the control group either, failing to support the notion that the slMFB is implicated in attentional abilities. Specific interest in the slMFB was indicated by the strong association between catecholamines and attention (for review see Clark & Noudoost, 2014), the lack of research into the slMFB in a TBI cohort to date, and the potential benefit of identifying individuals who may benefit from pharmacological interventions known to moderate catecholamines within the PFC, such as methylphenidate. Additionally, other research has found associations between white matter microstructural alterations within a single pathway and complex cognitive abilities. For example, Johnson et al. (2011) found that reduced FA in the uncinate fasciculus predicted emotional and behavioral regulation problems in children following TBI.

With regard to performance on attentional tasks, TBI participants were found to perform more poorly on cognitive tasks when compared to controls. They demonstrated slowed information processing speed on tasks including the TMT‐A, SAT, *n*‐back, Hayling and SDMT relative to the control group. As the majority of these tasks contain a motor component, it is difficult to determine if the TBI group demonstrated cognitive processing speed deficits alone, if it was a combination of both motor and cognitive slowing, or if the results reflect a slowing in motor speed only. The Hayling task, however, does not contain a motor component, and thus is not reliant on motor speed, providing evidence for the presence of cognitive information processing speed deficits within the TBI group. This corroborates previous literature identifying slowed information processing speed as a major outcome following TBI (Felmingham, Baguley, & Green, 2004; Willmott et al., 2009). Interestingly, after applying the Bonferroni correction, no significant difference was found between the two groups with regard to time to complete TMT‐B. This is in contrast with previous findings (Spitz et al. (2012)) and raises the possibility that the TBI group were only impaired on basic cognitive tasks (e.g., TMT‐A). However, given participants with TBI demonstrated reduced processing speed on the SAT and *n*‐back, two tasks carrying a high cognitive load, this is unlikely to be the case.

Consistent with previous research (Ponsford & Kinsella, 1992; Willmott et al., 2009), no difference was found between the TBI and control group on Hayling, TMT, SDMT, or Ruff2&7 in terms of accuracy, suggesting participants with TBI may have been sacrificing speed for accuracy on these tasks. With regard to working memory, no significant difference was found between groups on any condition of Digit Span. Although working memory deficits have been identified following TBI (Willmott et al., 2009), other studies have also failed to differentiate between participants with TBI and controls on this task (Draper & Ponsford, 2008). Additionally, number of errors on all conditions of the *n*‐back was comparable for the TBI and control group, indicating intact working memory. Similarly, no significant differences were found between groups with regard to the number of errors or misses on any condition of the SAT. Previous literature has demonstrated individuals with TBI make increased errors and misses on the CSAT, but not the SSAT of the SAT, when compared to controls. This is believed to reflect deficits in selective attention (Ziino & Ponsford, 2006). The current findings are inconsistent with this, suggesting intact selective attention. Overall, the prominent finding within the TBI group was slowed processing speed, with little evidence for strategic control of attention deficits.

The current findings are consistent with previous research that has suggested slowed information processing speed may largely account for attention deficits following TBI (Dymowski, Owens, Ponsford, & Willmott, 2015; Felmingham et al., 2004; Mathias & Wheaton, 2007). Other research utilizing more complex tasks carrying a higher working memory load (i.e., dual tasks experiments with multiple dual conditions of varying difficulty) has demonstrated strategic control of attention deficits not accounted for by processing speed (Asloun et al., 2008; Azouvi, Jokic, Der Linden, Marlier, & Bussel, 1996), suggesting our tasks may not have been sufficiently complex to capture higher level attention deficits.

### 4.1 Future research

It is possible the tasks used in this study were not sensitive to the type of deficits attributable to slMFB damage. Given the slMFB projects to the PFC, it is possible that this particular tract is associated with more executive aspects of attention, rather than basic processing speed. In addition, the slMFB is known to play a major role in both affective and addiction disorders due to its implication in the brain reward systems (Alcaro & Panksepp, 2011; Coenen et al., 2009, 2012; Wise, 2005). Thus, future research using tasks such as the IOWA Gambling Task (Bechara, Damasio, Damasio, & Anderson, 1994), which encompasses a reward‐based learning component (Fellows, 2004), may be more effective in elucidating the deficits and symptoms associated with damage to the slMFB.

### 4.2 Limitations

Although the sample was well controlled and well matched, it was relatively small, possibly missing significant associations and leading to Type II error. Additionally, given the TBI group consisted of mainly moderate to severe injuries, it is likely the majority of white matter tracts would exhibit white matter microstructure alterations to some extent. As this study only investigated the slMFB, however, it is unknown whether the slMFB was more or less damaged than any other white matter tract within the brain. Furthermore, although no significant associations were identified between performance on attention tasks and FA or MD within the slMFB, as the study did not investigate a comparison tract, the specific relationship (or lack thereof) between attention performance and slMFB changes following TBI is unknown. As previously mentioned, however, the aim of the study was to explore whether the slMFB was damaged post‐TBI and if it was associated with attentional performance, given its strong implication in the DA system. This was the first study to demonstrate that the slMFB is indeed, damaged following TBI. However, no association with attentional deficits was found in the current TBI cohort. Finally, it is important to note the potential influence of partial volume effects on the results (Alexander, Hasan, Lazar, Tsuruda, & Parker, 2001; Vos, Jones, Viergever, & Leemans, 2011). Given individuals with TBI usually demonstrate some degree of brain atrophy, and that the pathway investigated runs along the lateral wall of the ventricles, it is possible that the DTI metrics were contaminated by the inadvertent measurement of cerebrospinal fluid resulting in reduced FA and increased MD values. However, each individual scan was inspected and participants with problematic brain atrophy were excluded.

## 5 Conclusion

Attentional abilities have long been linked to DAergic activity. TBI is associated with disruptions to the DA system, which may contribute to attentional deficits. This is the first study to provide evidence of white matter damage to the slMFB following TBI, extending upon previous research demonstrating DA disruption following TBI. No association was found between attentional performance and slMFB microstructural alterations in either group, failing to provide evidence for the role of the slMFB in the attentional system. Investigating associations between outcomes such as impulsivity/inhibition or mood disturbance and slMFB microstructural alterations following TBI may provide further evidence of the role of the slMFB in cognition and the subsequent implications associated with damage to this pathway.

## Conflict of Interest

The authors declare that there is no conflict of interest. The authors alone are responsible for the content and preparation of the paper.
